# Notes and News

**Published:** 1902-12-06

**Authors:** 


### Notes and News.

The King, who is patron of the Middlesex Hospital, has sent the sum of £100 as an annual subscription to the hospital. This subscription is in continuance of the contribution which has been made by reigning sovereigns during the past 100 years.

Notice has been received that the petitions in favour of the separation of the Universities of Manchester and Liverpool will be heard by the Privy Council on December 17th.

The foundation-stone of a new sanatorium has been laid near Skipton for the Bradford Board of Guardians. This is the first consumption sanatorium established by Poor-law authorities. The building is to be of wood.

Dr. Robert Koch, the eminent bacteriologist and discoverer of the tubercle bacillus, has presented to the Committee of Management of the Mount Vernon Hospital for Consumption a portrait of himself, to be placed in the library of the new central out-patients' department in Fitzroy Square.

Sir Henry Burdett addressed a large meeting at Tunbridge Wells on November 29th on behalf of the League of Mercy. The Marquis of Camden, President of the League for Tunbridge Wells, occupied the chair. Sir Henry Burdett, besides advocating the needs of the London hospitals, appealed to the locality to improve their own hospital, which after having visited he found to be sadly in need of improvement.

At the St. Louis Universal Exposition to be held in 1904, importance will be attached to the public health section. Matters relating to sanitary legislation and investigation, the prevention of infectious diseases, vital statistics, and kindred subjects will find a place. Arrangements for the exposition are already advanced. The exposition will commemorate the acquisition of Louisiana territory by the United States. The city of St. Louis is interesting historically and otherwise, and the exhibition promises to be arranged on artistic lines.

Owing to funds being much needed for the Metropolitan Hospital, Kingsland Road, the Ladies' Association have decided to hold an advertisement ball at the Empress Rooms, Royal Palace Hotel, Kensington, on Tuesday, February 10th.

General pity must be felt for the inhabitants of a lodging house in Accrington who, numbering 100 persons, are shut up as suspects, cases of small-pox having occurred. We hope that exceptions were made in the case of any who could prove recent successful vaccination.

The Council of King's College have made the following appointments at King's College Hospital. In consequence of the resignation of the chair of clinical surgery at King's College by Professor Rose, Professor W. Watson Cheyne, C.B., F.R.S., has been elected to succeed him. Mr. A. Carless, M.B., F.R.C.S., has been elected Professor of Surgery, and Mr. F. Burghard, M.B., F.R.C.S., to be Teacher of Operative Surgery.

We often hear the just complaint that the burden of providing Christmas presents and entertainments in London falls too largely upon the medical and nursing staff of the hospitals. We think this might be altered if secretaries would follow the example of Mr. Howard Collins, Secretary to the Birmingham General Hospital, who issues a circular drawing attention to the needs of the patients at Christmas. Governors and subscribers to the London Hospitals would, we feel sure, respond readily to an appeal to brighten the enforced residence of the sick poor in the hospitals at Christmas-time.

A novel experiment in the interest of dietetics is about to be made by the Agricultural Department at Washington. Twelve men are to be placed on a diet of adulterated and unadulterated food for six months. For two weeks at a time six men will live on adulterated food, and their weights and general health will be compared with those who have lived on pure food during the same period. The material for experiment has been readily found by the offer of free maintenance during the period of experiment. The adulterated foods will contain colouring matters, boric acid, and other chemicals introduced into foods in America.

The sale of surplus goods from the Imperial Coronation bazaar, and annual sale of work of "The Children's Salon," were held in the Helena Ward of the Hospital for Sick Children, Great Ormond Street, on Wednesday and Thursday, 3rd and 4th inst. H.R.H. the Princess Henry of Battenberg opened the sale on Wednesday at about 3.45 p.m., and went the round of the stalls, subsequently inspecting some of the wards, including the Alexandra Ward. The particular object of this sale is to assist in raising £1,000 for the endowment of the "Mary Victoria" Cot, named by permission of H.R.H. the Princess of Wales after her daughter. The cot was on view, over which was hanging a beautifully executed pastel portrait of Princess Mary Victoria of Wales, the gift of the artist, Mrs. Adrian Hope.

